# The vigorous immune microenvironment of microsatellite instable colon cancer is balanced by multiple counter-inhibitory checkpoints

**DOI:** 10.1186/2051-1426-3-S2-P410

**Published:** 2015-11-04

**Authors:** Nicolas L Losa, Michael Cruise, Ada Tam, Elizabeth Wick, Elizabeth Hechenbleikner, Janis M Taube, Richard Blosser, Hongni Fan, Hao Wang, Brandon Luber, Ming Zhang, Nickolas Papadopoulos, Kenneth Kinzler, Bert Vogelstein, Cynthia Sears, Robert A Anders, Drew Pardoll, Franck Housseau, Nicholas Siegel

**Affiliations:** 1 Johns Hopkins University School of Medicine, Baltimore, MD, USA; 2 Cleveland Clinic, Cleveland, OH, USA; 3 Johns Hopkins University: School of Medicine, Baltimore, MD, USA; 4 Johns Hopkins Hospital, Baltimore, MD, USA

## 

We examined the immune microenvironment of primary colorectal cancer (CRC) using immunohistochemistry, laser capture microdissection/qRT-PCR, flow cytometry and functional analysis of tumor infiltrating lymphocytes. A subset of CRC displayed high infiltration with activated CD8+ CTL as well as activated Th1 cells characterized by IFN-gamma production and the Th1 transcription factor Tbet. Parallel analysis of tumor genotypes revealed that virtually all of the tumors with this active Th1/CTL microenvironment had defects in mismatch repair, as evidenced by microsatellite instability (MSI). Counterbalancing this active Th1/CTL microenvironment, MSI tumors selectively demonstrated highly up-regulated expression of multiple immune checkpoints, including five - PD-1, PD-L1, CTLA-4, LAG-3 and IDO - currently being targeted clinically with inhibitors. These findings link tumor genotype with the immune microenvironment, and explain why MSI tumors are not naturally eliminated despite a hostile Th1/CTL microenvironment. They further suggest that blockade of specific checkpoints may be selectively efficacious in the MSI subset of CRC. Our findings are the first to demonstrate a link between a genetically defined subtype of cancer and its corresponding expression of immune checkpoints in the tumor microenvironment. The mismatch repair defective subset of CRC selectively up-regulates at least 5 checkpoint molecules that are targets of inhibitors currently being clinically tested. Furthermore, our results were clinically validated in a Phase II study at Hopkins which showed mismatch-repair status as a predictor of clinical benefit to immune checkpoint blockade with pembrolizumab.

